# Crystal structure of cafenstrole

**DOI:** 10.1107/S2056989015013869

**Published:** 2015-07-29

**Authors:** Gihaeng Kang, Jineun Kim, Hyunjin Park, Tae Ho Kim

**Affiliations:** a Department of Chemistry and Research Institute of Natural Sciences, Gyeongsang National University, Jinju 660-701, Republic of Korea

**Keywords:** crystal structure, cafenstrole, triazole, herbicide,

## Abstract

The title compound (systematic name: *N*,*N*-diethyl-3-mesitylsulfonyl-1*H*-1,2,4-triazole-1-carboxamide), C_16_H_22_N_4_O_3_S, is a triazole herbicide. The dihedral angle between the planes of the triazole and benzene ring planes is 88.14 (10)°. In the crystal, C—H⋯O hydrogen bonds and weak C—H⋯π inter­actions link adjacent mol­ecules, forming one-dimensional chains along the *a* axis.

## Related literature   

For information on the herbicidal properties of the title compound, see: Takahashi *et al.* (2001 ▸). For related crystal structure, see: Ohkata *et al.* (2002 ▸).
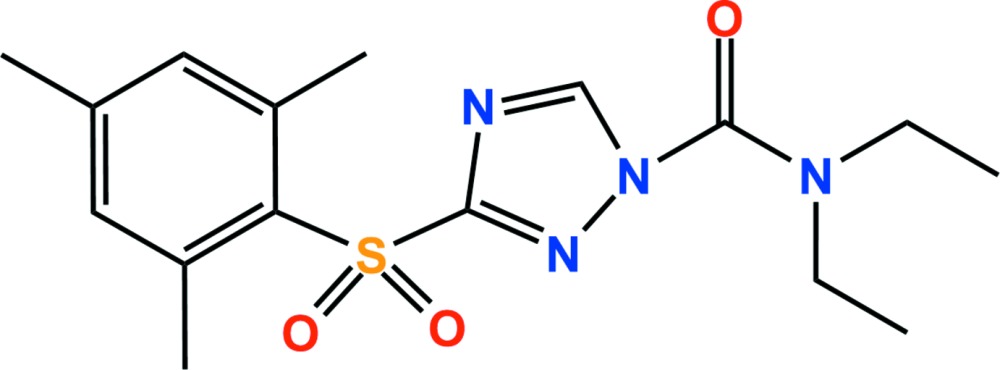


## Experimental   

### Crystal data  

C_16_H_22_N_4_O_3_S
*M* *_r_* = 350.43
Monoclinic, 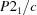

*a* = 7.2800 (3) Å
*b* = 8.0410 (4) Å
*c* = 30.1792 (13) Å
β = 95.290 (3)°
*V* = 1759.12 (14) Å^3^
*Z* = 4
Mo *K*α radiation
μ = 0.21 mm^−1^
*T* = 173 K
0.36 × 0.30 × 0.02 mm

### Data collection  

Bruker APEXII CCD diffractometer
Absorption correction: multi-scan (*SADABS*; Bruker, 2013 ▸) *T*_min_ = 0.929, *T*_max_ = 0.996
11871 measured reflections
3385 independent reflections
2760 reflections with *I* > 2σ(*I*)
*R*_int_ = 0.033

### Refinement  

*R*[*F* ^2^ > 2σ(*F* ^2^)] = 0.051
*wR*(*F* ^2^) = 0.118
*S* = 1.12
3385 reflections
222 parameters
H-atom parameters constrained
Δρ_max_ = 0.32 e Å^−3^
Δρ_min_ = −0.35 e Å^−3^

### 

Data collection: *APEX2* (Bruker, 2013 ▸); cell refinement: *SAINT* (Bruker, 2013 ▸); data reduction: *SAINT*; program(s) used to solve structure: *SHELXS97* (Sheldrick, 2008 ▸); program(s) used to refine structure: *SHELXL2013* (Sheldrick, 2015 ▸); molecular graphics: *DIAMOND* (Brandenburg, 2010 ▸); software used to prepare material for publication: *SHELXTL* (Sheldrick, 2008 ▸).

## Supplementary Material

Crystal structure: contains datablock(s) global, I. DOI: 10.1107/S2056989015013869/hg5453sup1.cif

Structure factors: contains datablock(s) I. DOI: 10.1107/S2056989015013869/hg5453Isup2.hkl

Supporting information file. DOI: 10.1107/S2056989015013869/hg5453Isup3.cml

**Fig. 1.**
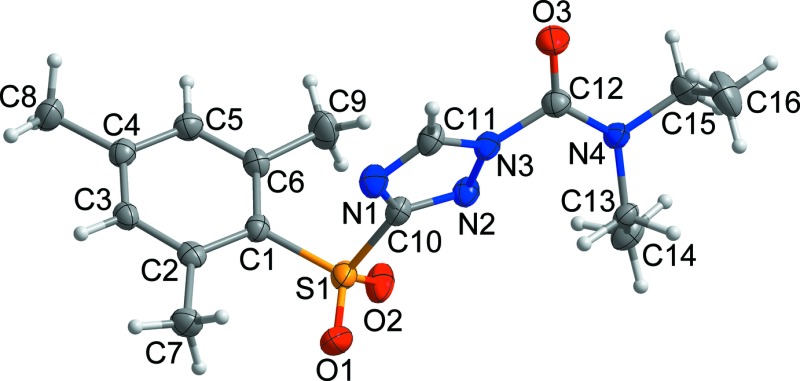
The asymmetric unit of the title compound with the atom numbering scheme. Displacement ellipsoids are drawn at the 50% probability level. H atoms are shown as small spheres of arbitrary radius.

**Fig. 2.**
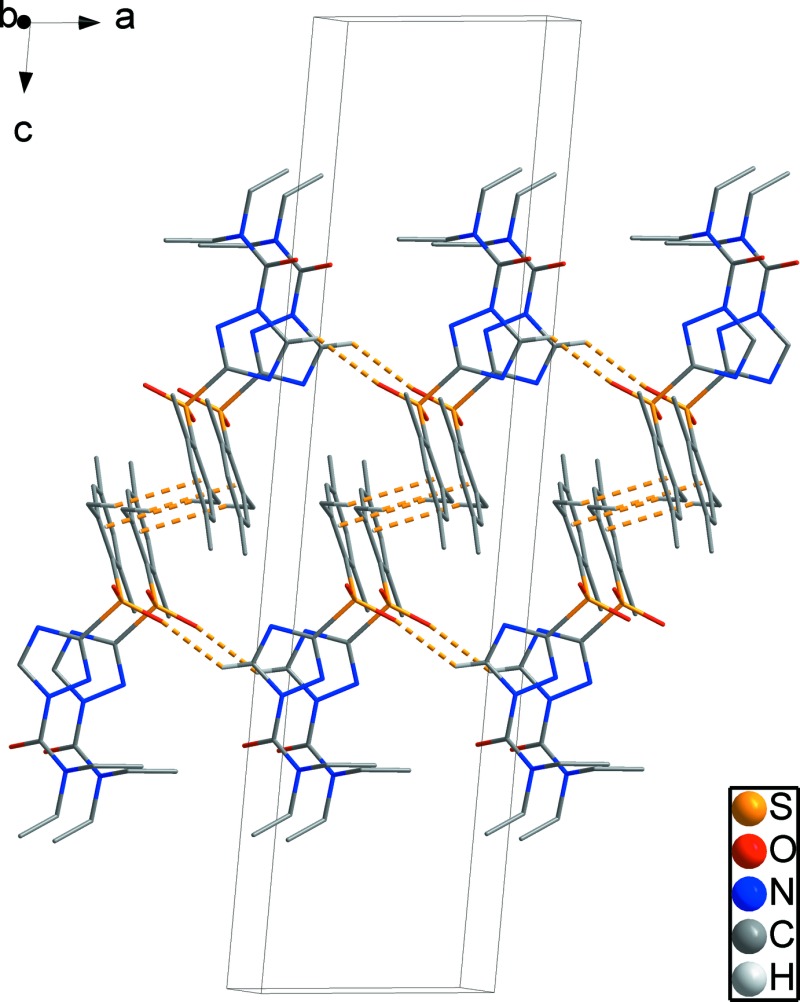
Crystal packing viewed along the b axis. The intermolecular interactions are shown as dashed lines.

. DOI: 10.1107/S2056989015013869/hg5453fig1.tif
The asymmetric unit of the title compound with the atom numbering scheme. Displacement ellipsoids are drawn at the 50% probability level. H atoms are shown as small spheres of arbitrary radius.

b . DOI: 10.1107/S2056989015013869/hg5453fig2.tif
Crystal packing viewed along the *b* axis. The inter­molecular inter­actions are shown as dashed lines.

CCDC reference: 1414616

Additional supporting information:  crystallographic information; 3D view; checkCIF report

## Figures and Tables

**Table 1:** Hydrogen-bond geometry (, )

*D*H*A*	*D*H	H*A*	*D* *A*	*D*H*A*
C11H11O2^i^	0.95	2.38	3.136(3)	136
C7H7*C* *Cg*1^ii^	0.98	2.80	3.561(3)	135

Symmetry codes: (i) 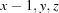; (ii) 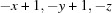.

## supplementary crystallographic information

### S1. Comment

Cafenstrole, or *N,N*-diethyl-3-mesitylsulfonyl-1*H*-1,2,4-triazole-1-carboxamide, is a triazole herbicide and has been used for rice cultivation which especially inhibits the germination of grass weeds (Takahashi *et al.*, 2001). However, until now its crystal structure has not been reported. In the title compound (Fig. 1), the dihedral angle between the planes of the triazole ring and the phenyl ring planes is 88.14 (10)°. All bond lengths and bond angles are normal and comparable to those observed in similar crystal structure (Ohkata *et al.*, 2002).

In the crystal structure (Fig. 2, Table 1), C11—H11···O2 hydrogen bonds and weak intermolecular C7—H7C···*Cg*1 (*Cg*1 is the centroid of the C1–C6 ring) interactions link adjacent molecules, forming one-dimensional chains along to *a*-axis.

### S2. Experimental

The title compound was purchased from the Dr. Ehrenstorfer GmbH Company. Slow evaporation of a solution in CH_3_CN gave single crystals suitable for X-ray analysis.

### S3. Refinement

All H-atoms were positioned geometrically and refined using a riding model with d(C—H) = 0.98 Å, *U*_iso_ = 1.5*U*_eq_(C) for methyl group, d(C—H) = 0.99 Å, *U*_iso_ = 1.2*U*_eq_(C) for C*sp*^3^—H, d(C—H) = 0.95 Å, *U*_iso_ = 1.2*U*_eq_(C) for aromatic C—H.

### Crystal data

**Table:**

C_16_H_22_N_4_O_3_S	*D*_x_ = 1.323 Mg m^−^^3^
*M**_r_* = 350.43	Melting point: 390 K
Monoclinic, *P*2_1_/*c*	Mo *K*α radiation, λ = 0.71073 Å
*a* = 7.2800 (3) Å	Cell parameters from 3608 reflections
*b* = 8.0410 (4) Å	θ = 2.6–25.8°
*c* = 30.1792 (13) Å	µ = 0.21 mm^−^^1^
β = 95.290 (3)°	*T* = 173 K
*V* = 1759.12 (14) Å^3^	Plate, colourless
*Z* = 4	0.36 × 0.30 × 0.02 mm
*F*(000) = 744	

### Data collection

**Table:**

Bruker APEXII CCD diffractometer	2760 reflections with *I* > 2σ(*I*)
φ and ω scans	*R*_int_ = 0.033
Absorption correction: multi-scan (*SADABS*; Bruker, 2013)	θ_max_ = 26.0°, θ_min_ = 1.4°
*T*_min_ = 0.929, *T*_max_ = 0.996	*h* = −8→7
11871 measured reflections	*k* = −9→9
3385 independent reflections	*l* = −37→37

### Refinement

**Table:**

Refinement on *F*^2^	0 restraints
Least-squares matrix: full	Hydrogen site location: inferred from neighbouring sites
*R*[*F*^2^ > 2σ(*F*^2^)] = 0.051	H-atom parameters constrained
*wR*(*F*^2^) = 0.118	*w* = 1/[σ^2^(*F*_o_^2^) + (0.0373*P*)^2^ + 1.5378*P*] where *P* = (*F*_o_^2^ + 2*F*_c_^2^)/3
*S* = 1.12	(Δ/σ)_max_ < 0.001
3385 reflections	Δρ_max_ = 0.32 e Å^−^^3^
222 parameters	Δρ_min_ = −0.35 e Å^−^^3^

### Special details

**Table:**

Geometry. All e.s.d.'s (except the e.s.d. in the dihedral angle between two l.s. planes) are estimated using the full covariance matrix. The cell e.s.d.'s are taken into account individually in the estimation of e.s.d.'s in distances, angles and torsion angles; correlations between e.s.d.'s in cell parameters are only used when they are defined by crystal symmetry. An approximate (isotropic) treatment of cell e.s.d.'s is used for estimating e.s.d.'s involving l.s. planes.

### Fractional atomic coordinates and isotropic or equivalent isotropic displacement parameters (Å^2^)

**Table:**

	*x*	*y*	*z*	*U*_iso_*/*U*_eq_	
S1	0.41370 (9)	0.41398 (9)	0.10060 (2)	0.03098 (19)	
O1	0.3991 (3)	0.2440 (2)	0.08694 (6)	0.0440 (5)	
O2	0.5862 (2)	0.4698 (3)	0.12191 (6)	0.0426 (5)	
O3	−0.0331 (3)	0.6067 (3)	0.24940 (6)	0.0450 (5)	
N1	0.0629 (3)	0.4287 (3)	0.12755 (6)	0.0321 (5)	
N2	0.2960 (3)	0.4724 (3)	0.18146 (6)	0.0280 (5)	
N3	0.1290 (3)	0.4783 (3)	0.19847 (6)	0.0262 (5)	
N4	0.2305 (3)	0.4731 (3)	0.27538 (6)	0.0265 (5)	
C1	0.3372 (3)	0.5499 (3)	0.05661 (7)	0.0243 (5)	
C2	0.2701 (3)	0.4890 (3)	0.01447 (7)	0.0250 (5)	
C3	0.2198 (3)	0.6048 (3)	−0.01847 (7)	0.0262 (5)	
H3	0.1760	0.5657	−0.0472	0.031*	
C4	0.2303 (3)	0.7740 (3)	−0.01148 (8)	0.0275 (6)	
C5	0.2917 (3)	0.8308 (3)	0.03064 (8)	0.0293 (6)	
H5	0.2964	0.9472	0.0360	0.035*	
C6	0.3468 (3)	0.7227 (3)	0.06518 (7)	0.0272 (6)	
C7	0.2494 (4)	0.3081 (3)	0.00170 (8)	0.0348 (6)	
H7A	0.1922	0.2994	−0.0289	0.052*	
H7B	0.1713	0.2521	0.0219	0.052*	
H7C	0.3711	0.2552	0.0038	0.052*	
C8	0.1789 (4)	0.8928 (4)	−0.04883 (8)	0.0389 (7)	
H8A	0.2902	0.9482	−0.0574	0.058*	
H8B	0.0931	0.9763	−0.0391	0.058*	
H8C	0.1199	0.8317	−0.0744	0.058*	
C9	0.4143 (4)	0.7991 (4)	0.10936 (8)	0.0456 (8)	
H9A	0.5462	0.7758	0.1159	0.068*	
H9B	0.3459	0.7514	0.1328	0.068*	
H9C	0.3947	0.9196	0.1081	0.068*	
C10	0.2465 (3)	0.4418 (3)	0.13928 (7)	0.0263 (5)	
C11	−0.0062 (3)	0.4550 (3)	0.16559 (8)	0.0302 (6)	
H11	−0.1343	0.4573	0.1694	0.036*	
C12	0.1025 (3)	0.5235 (3)	0.24394 (8)	0.0293 (6)	
C13	0.3683 (3)	0.3424 (3)	0.27089 (8)	0.0253 (5)	
H13A	0.3367	0.2817	0.2427	0.030*	
H13B	0.3635	0.2621	0.2956	0.030*	
C14	0.5623 (3)	0.4096 (4)	0.27125 (9)	0.0364 (6)	
H14A	0.5731	0.4752	0.2443	0.055*	
H14B	0.6499	0.3169	0.2723	0.055*	
H14C	0.5896	0.4803	0.2975	0.055*	
C15	0.2014 (4)	0.5244 (4)	0.32107 (8)	0.0371 (7)	
H15A	0.1512	0.6389	0.3205	0.045*	
H15B	0.3214	0.5253	0.3394	0.045*	
C16	0.0700 (4)	0.4095 (5)	0.34227 (9)	0.0528 (9)	
H16A	−0.0498	0.4096	0.3245	0.079*	
H16B	0.0542	0.4480	0.3725	0.079*	
H16C	0.1204	0.2964	0.3435	0.079*	

### Atomic displacement parameters (Å^2^)

**Table:**

	*U*^11^	*U*^22^	*U*^33^	*U*^12^	*U*^13^	*U*^23^
S1	0.0289 (4)	0.0379 (4)	0.0261 (3)	0.0080 (3)	0.0024 (2)	0.0056 (3)
O1	0.0604 (13)	0.0328 (11)	0.0394 (10)	0.0158 (10)	0.0082 (9)	0.0063 (9)
O2	0.0237 (10)	0.0704 (15)	0.0327 (9)	0.0075 (10)	−0.0024 (7)	0.0098 (10)
O3	0.0377 (11)	0.0587 (14)	0.0388 (10)	0.0233 (10)	0.0043 (8)	0.0019 (10)
N1	0.0265 (11)	0.0399 (14)	0.0290 (10)	−0.0012 (10)	−0.0020 (9)	0.0017 (10)
N2	0.0220 (11)	0.0345 (12)	0.0275 (10)	0.0021 (9)	0.0020 (8)	0.0046 (10)
N3	0.0213 (11)	0.0306 (12)	0.0264 (10)	0.0019 (9)	0.0000 (8)	0.0035 (9)
N4	0.0290 (11)	0.0244 (11)	0.0251 (10)	0.0033 (9)	−0.0031 (8)	−0.0011 (9)
C1	0.0214 (12)	0.0284 (13)	0.0230 (11)	0.0013 (11)	0.0018 (9)	0.0029 (11)
C2	0.0173 (12)	0.0319 (14)	0.0261 (11)	−0.0003 (10)	0.0037 (9)	−0.0022 (11)
C3	0.0241 (12)	0.0326 (14)	0.0215 (11)	0.0007 (11)	0.0003 (9)	−0.0030 (11)
C4	0.0233 (13)	0.0316 (14)	0.0276 (12)	0.0018 (11)	0.0026 (10)	0.0034 (11)
C5	0.0321 (14)	0.0252 (13)	0.0309 (12)	−0.0004 (11)	0.0043 (11)	−0.0005 (11)
C6	0.0272 (13)	0.0318 (14)	0.0226 (11)	−0.0017 (11)	0.0016 (10)	−0.0004 (11)
C7	0.0390 (16)	0.0295 (15)	0.0353 (13)	0.0013 (13)	0.0002 (11)	−0.0050 (12)
C8	0.0479 (17)	0.0350 (16)	0.0328 (13)	0.0050 (14)	−0.0023 (12)	0.0057 (13)
C9	0.066 (2)	0.0384 (17)	0.0306 (14)	−0.0064 (16)	−0.0051 (13)	−0.0052 (13)
C10	0.0267 (13)	0.0270 (13)	0.0246 (11)	0.0018 (11)	−0.0011 (10)	0.0046 (11)
C11	0.0256 (13)	0.0338 (15)	0.0299 (12)	0.0007 (11)	−0.0034 (10)	0.0067 (12)
C12	0.0253 (14)	0.0319 (14)	0.0306 (13)	0.0029 (12)	0.0022 (10)	0.0014 (11)
C13	0.0276 (13)	0.0210 (12)	0.0265 (11)	0.0040 (10)	−0.0014 (10)	0.0047 (10)
C14	0.0278 (14)	0.0360 (15)	0.0441 (15)	−0.0003 (12)	−0.0026 (11)	0.0088 (13)
C15	0.0461 (17)	0.0354 (15)	0.0281 (13)	0.0066 (13)	−0.0057 (12)	−0.0099 (12)
C16	0.0481 (18)	0.083 (3)	0.0285 (13)	−0.0110 (18)	0.0087 (13)	−0.0122 (16)

### Geometric parameters (Å, º)

**Table:**

S1—O1	1.429 (2)	C6—C9	1.509 (3)
S1—O2	1.4293 (19)	C7—H7A	0.9800
S1—C1	1.769 (2)	C7—H7B	0.9800
S1—C10	1.777 (2)	C7—H7C	0.9800
O3—C12	1.216 (3)	C8—H8A	0.9800
N1—C11	1.313 (3)	C8—H8B	0.9800
N1—C10	1.355 (3)	C8—H8C	0.9800
N2—C10	1.314 (3)	C9—H9A	0.9800
N2—N3	1.364 (3)	C9—H9B	0.9800
N3—C11	1.345 (3)	C9—H9C	0.9800
N3—C12	1.450 (3)	C11—H11	0.9500
N4—C12	1.331 (3)	C13—C14	1.511 (3)
N4—C13	1.468 (3)	C13—H13A	0.9900
N4—C15	1.473 (3)	C13—H13B	0.9900
C1—C2	1.408 (3)	C14—H14A	0.9800
C1—C6	1.414 (4)	C14—H14B	0.9800
C2—C3	1.387 (3)	C14—H14C	0.9800
C2—C7	1.509 (3)	C15—C16	1.513 (4)
C3—C4	1.378 (4)	C15—H15A	0.9900
C3—H3	0.9500	C15—H15B	0.9900
C4—C5	1.385 (3)	C16—H16A	0.9800
C4—C8	1.499 (3)	C16—H16B	0.9800
C5—C6	1.387 (3)	C16—H16C	0.9800
C5—H5	0.9500		
			
O1—S1—O2	118.01 (13)	H8A—C8—H8C	109.5
O1—S1—C1	111.37 (11)	H8B—C8—H8C	109.5
O2—S1—C1	110.25 (12)	C6—C9—H9A	109.5
O1—S1—C10	105.83 (12)	C6—C9—H9B	109.5
O2—S1—C10	106.94 (11)	H9A—C9—H9B	109.5
C1—S1—C10	103.17 (11)	C6—C9—H9C	109.5
C11—N1—C10	101.98 (19)	H9A—C9—H9C	109.5
C10—N2—N3	101.33 (18)	H9B—C9—H9C	109.5
C11—N3—N2	109.55 (19)	N2—C10—N1	116.4 (2)
C11—N3—C12	125.5 (2)	N2—C10—S1	121.10 (18)
N2—N3—C12	124.48 (19)	N1—C10—S1	122.51 (17)
C12—N4—C13	126.3 (2)	N1—C11—N3	110.7 (2)
C12—N4—C15	115.5 (2)	N1—C11—H11	124.6
C13—N4—C15	116.46 (19)	N3—C11—H11	124.6
C2—C1—C6	120.9 (2)	O3—C12—N4	126.5 (2)
C2—C1—S1	121.47 (19)	O3—C12—N3	116.5 (2)
C6—C1—S1	117.58 (17)	N4—C12—N3	116.9 (2)
C3—C2—C1	117.4 (2)	N4—C13—C14	112.9 (2)
C3—C2—C7	116.8 (2)	N4—C13—H13A	109.0
C1—C2—C7	125.8 (2)	C14—C13—H13A	109.0
C4—C3—C2	123.1 (2)	N4—C13—H13B	109.0
C4—C3—H3	118.4	C14—C13—H13B	109.0
C2—C3—H3	118.4	H13A—C13—H13B	107.8
C3—C4—C5	118.3 (2)	C13—C14—H14A	109.5
C3—C4—C8	120.5 (2)	C13—C14—H14B	109.5
C5—C4—C8	121.1 (2)	H14A—C14—H14B	109.5
C4—C5—C6	122.0 (2)	C13—C14—H14C	109.5
C4—C5—H5	119.0	H14A—C14—H14C	109.5
C6—C5—H5	119.0	H14B—C14—H14C	109.5
C5—C6—C1	118.2 (2)	N4—C15—C16	112.1 (2)
C5—C6—C9	117.2 (2)	N4—C15—H15A	109.2
C1—C6—C9	124.6 (2)	C16—C15—H15A	109.2
C2—C7—H7A	109.5	N4—C15—H15B	109.2
C2—C7—H7B	109.5	C16—C15—H15B	109.2
H7A—C7—H7B	109.5	H15A—C15—H15B	107.9
C2—C7—H7C	109.5	C15—C16—H16A	109.5
H7A—C7—H7C	109.5	C15—C16—H16B	109.5
H7B—C7—H7C	109.5	H16A—C16—H16B	109.5
C4—C8—H8A	109.5	C15—C16—H16C	109.5
C4—C8—H8B	109.5	H16A—C16—H16C	109.5
H8A—C8—H8B	109.5	H16B—C16—H16C	109.5
C4—C8—H8C	109.5		
			
C10—N2—N3—C11	−1.4 (3)	N3—N2—C10—S1	−177.87 (17)
C10—N2—N3—C12	−174.3 (2)	C11—N1—C10—N2	0.7 (3)
O1—S1—C1—C2	0.7 (2)	C11—N1—C10—S1	178.95 (19)
O2—S1—C1—C2	133.7 (2)	O1—S1—C10—N2	113.2 (2)
C10—S1—C1—C2	−112.4 (2)	O2—S1—C10—N2	−13.4 (2)
O1—S1—C1—C6	−178.97 (18)	C1—S1—C10—N2	−129.7 (2)
O2—S1—C1—C6	−46.0 (2)	O1—S1—C10—N1	−65.0 (2)
C10—S1—C1—C6	67.9 (2)	O2—S1—C10—N1	168.3 (2)
C6—C1—C2—C3	2.1 (3)	C1—S1—C10—N1	52.1 (2)
S1—C1—C2—C3	−177.58 (17)	C10—N1—C11—N3	−1.5 (3)
C6—C1—C2—C7	−178.5 (2)	N2—N3—C11—N1	2.0 (3)
S1—C1—C2—C7	1.8 (3)	C12—N3—C11—N1	174.8 (2)
C1—C2—C3—C4	−1.0 (4)	C13—N4—C12—O3	164.5 (3)
C7—C2—C3—C4	179.6 (2)	C15—N4—C12—O3	0.2 (4)
C2—C3—C4—C5	−0.9 (4)	C13—N4—C12—N3	−17.0 (4)
C2—C3—C4—C8	178.2 (2)	C15—N4—C12—N3	178.7 (2)
C3—C4—C5—C6	1.7 (4)	C11—N3—C12—O3	−29.9 (4)
C8—C4—C5—C6	−177.4 (2)	N2—N3—C12—O3	141.9 (3)
C4—C5—C6—C1	−0.6 (4)	C11—N3—C12—N4	151.5 (2)
C4—C5—C6—C9	178.9 (2)	N2—N3—C12—N4	−36.8 (3)
C2—C1—C6—C5	−1.3 (4)	C12—N4—C13—C14	110.4 (3)
S1—C1—C6—C5	178.35 (18)	C15—N4—C13—C14	−85.5 (3)
C2—C1—C6—C9	179.2 (2)	C12—N4—C15—C16	82.9 (3)
S1—C1—C6—C9	−1.2 (3)	C13—N4—C15—C16	−83.0 (3)
N3—N2—C10—N1	0.4 (3)		

### Hydrogen-bond geometry (Å, º)

**Table:**

*D*—H···*A*	*D*—H	H···*A*	*D*···*A*	*D*—H···*A*
C11—H11···O2^i^	0.95	2.38	3.136 (3)	136
C7—H7*C*···*Cg*1^ii^	0.98	2.80	3.561 (3)	135

Symmetry codes: (i) *x*−1, *y*, *z*; (ii) −*x*+1, −*y*+1, −*z*.
