# Supplementary figures and images for: Crystal structure of cafenstrole
Source: Acta Crystallogr E Crystallogr Commun. 2015 Jul 29;71(Pt 8):o614. doi: 10.1107/S2056989015013869 (PMC4571425; doi:10.1107/S2056989015013869)

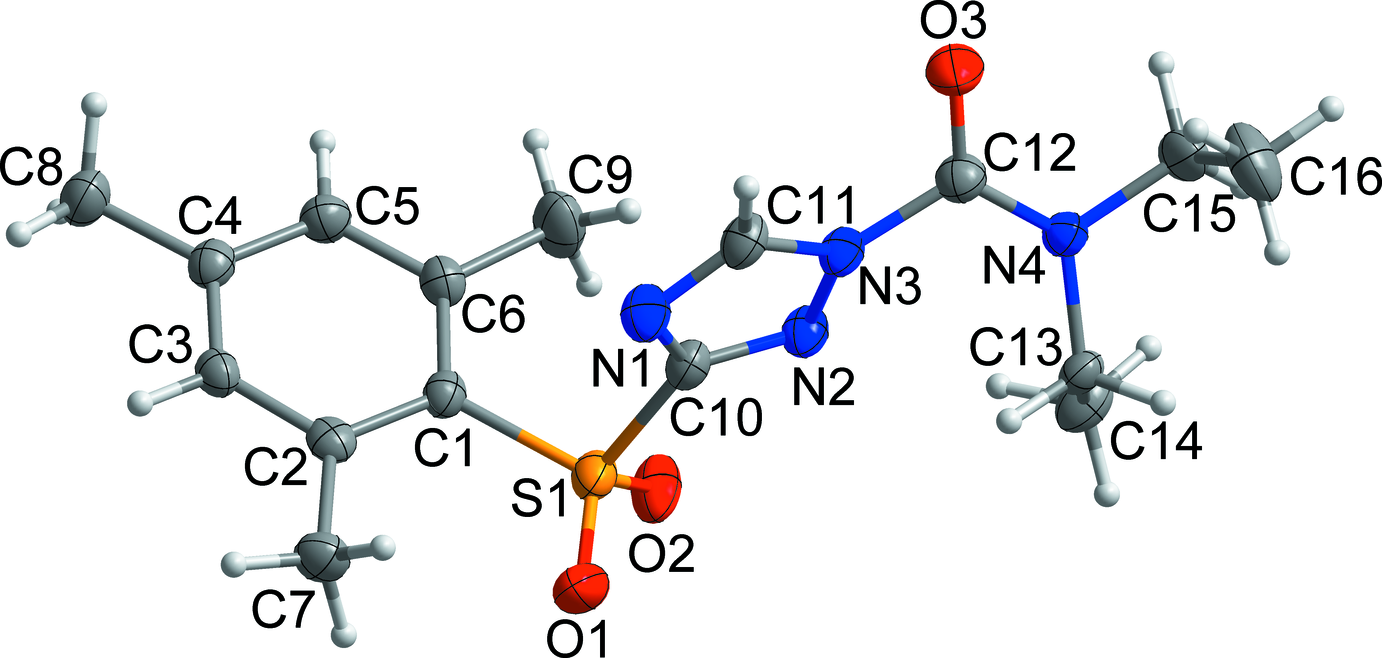

Supplement: Supplementary file 4 [file e-71-0o614-fig1.tif]

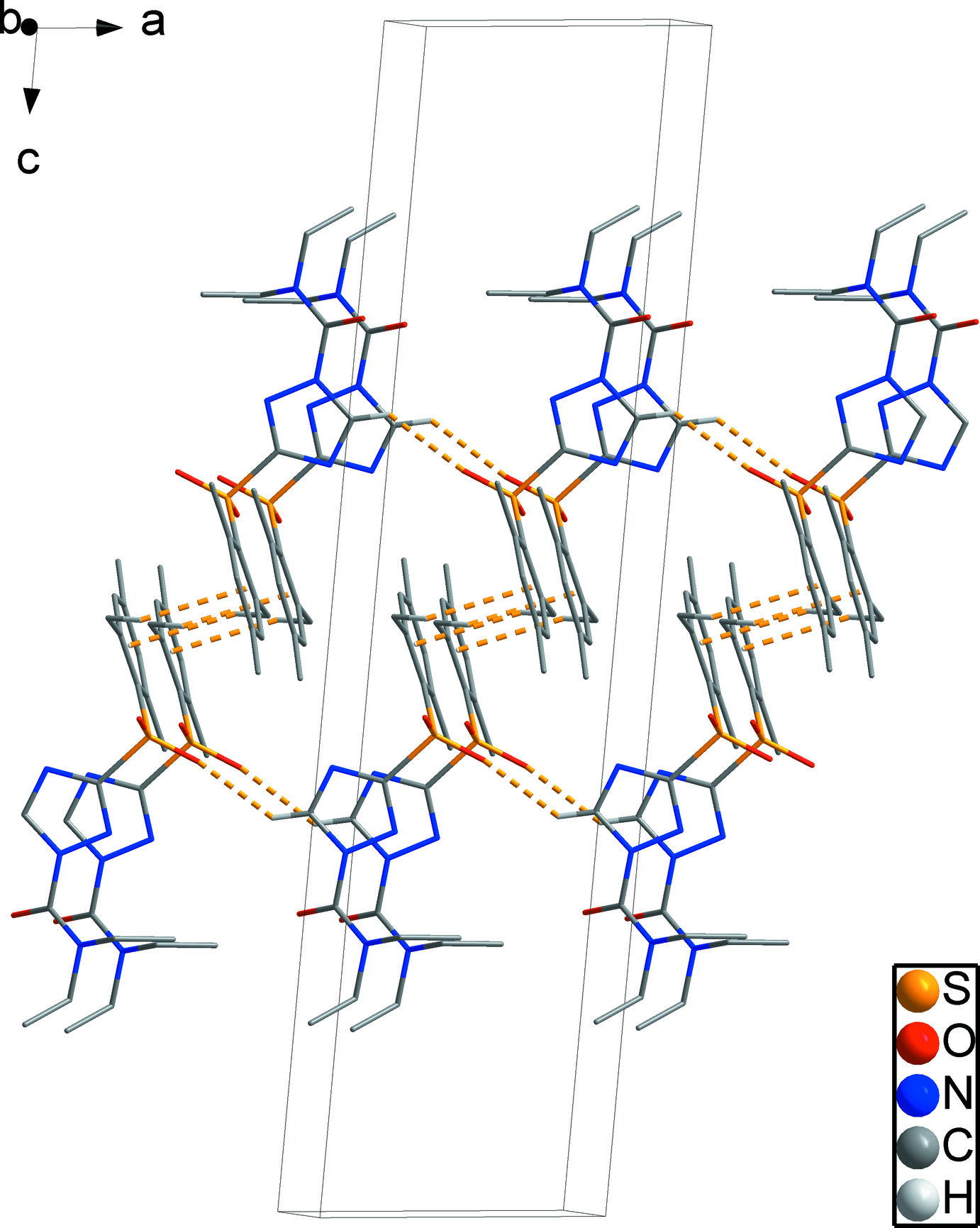

Supplement: Supplementary file 5 [file e-71-0o614-fig2.tif]
